# Fish can show emotional fever: stress-induced hyperthermia in zebrafish

**DOI:** 10.1098/rspb.2015.2266

**Published:** 2015-11-22

**Authors:** Sonia Rey, Felicity A. Huntingford, Sebastian Boltaña, Reynaldo Vargas, Toby G. Knowles, Simon Mackenzie

**Affiliations:** 1 Institute of Aquaculture, School of Natural Sciences, University of Stirling, Stirling FK9 4LA, UK; 2 Institut de Biotecnologia i Biomedicina, Universitat Autònoma de Barcelona, Bellaterra, Spain; 3 School of Veterinary Science, University of Bristol, Langford, Bristol BS40 5DU, UK

**Keywords:** zebrafish, consciousness, stress-induced hyperthermia, emotional fever, fish sentience, fish welfare

## Abstract

Whether fishes are sentient beings remains an unresolved and controversial question. Among characteristics thought to reflect a low level of sentience in fishes is an inability to show stress-induced hyperthermia (SIH), a transient rise in body temperature shown in response to a variety of stressors. This is a real fever response, so is often referred to as ‘emotional fever’. It has been suggested that the capacity for emotional fever evolved only in amniotes (mammals, birds and reptiles), in association with the evolution of consciousness in these groups. According to this view, lack of emotional fever in fishes reflects a lack of consciousness. We report here on a study in which six zebrafish groups with access to a temperature gradient were either left as undisturbed controls or subjected to a short period of confinement. The results were striking: compared to controls, stressed zebrafish spent significantly more time at higher temperatures, achieving an estimated rise in body temperature of about 2–4°C. Thus, zebrafish clearly have the capacity to show emotional fever. While the link between emotion and consciousness is still debated, this finding removes a key argument for lack of consciousness in fishes.

## 1. Background

The question of whether fishes are conscious, sentient beings remains unresolved and controversial. Those who argue that this is not the case [1–3] do so on the basis of the fact that the brain of fishes is relatively small and simple, lacking the cerebral cortex that mediates much high-level information processing in mammals. According to this view, fishes have little capacity for learning and memory and a very simple behavioural repertoire. They lack the cognitive ability to experience suffering and their responses to adverse circumstances, while perhaps more than straightforward reflexes, are still very simple and have little or no emotional content [3].

Others contest this view (e.g. [4–7]), pointing out that, although the fish brain is indeed smaller and organized differently from that of, say, a mammal, detailed morphological and behavioural analyses have highlighted structural homology and functional equivalence between forebrain structures in fishes and other vertebrates. Of particular relevance are the mammalian amygdala (involved in generation of emotions) and hippocampus (involved in learning and spatial memory), which are homologous and functionally equivalent to the dorsomedial and dorsolateral telencephalic pallium of the fish forebrain [8]. In addition, functional similarities with the mammalian limbic system have recently been indicated by the detection of increased levels of cortisol and serotonin in both these brain regions in Nile Tilapia (*Oreochromis niloticus*) after exposed to a stress situation (confinement) [9]. Concerning how fishes respond to noxious stimuli, a series of experimental studies by Braithwaite and colleagues ([10,11]; see [6,7] for reviews) has shown not only that fishes have the capacity to perceive noxious stimuli that induce pain in mammals, but that their responses to such stimuli involve physiological arousal, performance of stereotypical movements, changes in motivational state and quite complex attention shifts. These findings indicate that noxious stimuli induce shifts in fish mental states that go far beyond simple reflexes.

The recent literature provides numerous examples of the learning capacity of fishes [12] and of complex things that fishes do (reviewed in [6,7]). To give just a few examples, many fishes form mental maps of their environment and use these to perform complicated feats of navigation [13,14]. Males of the cichlid species *Astatotilapia burtini* can infer the relative social rank of territorial neighbours by observation, using a capacity for transitive inference (deducing that if A > B and B > C, then A must be greater than C. [15]), together with the ability to recognize and remember individual companions. Some fishes make use of tools, taken as an indication of a degree of consciousness in other groups. For example, wrasse use an anvil to break hard pellets that are too large to eat [16] while captive cod marked dorsally with a beaded tag learned to hook the tag onto the pulley mechanism of a demand feeder to obtain food [17].

Reviews on cognitive abilities and their relation on emotion and consciousness in fishes and across species have been published recently [18–20]. Emotion in this study will be understood as a causative state where an internal, central state is triggered by a specific stimulus and this can be externally observed by behavioural changes as well as by their associated cognitive, somatic and physiological responses. Emotions are different from simple stimulus response reflexes by the fact that the behaviour, or associated state variables, outlast the stimuli that elicit them [18,21]. Consciousness states are multidimensional and so are consciousness definitions of these states. Five are especially salient and these include the sleeping/awaking states, perceptual awareness and sensitivity, access versus phenomenal consciousness and self-awareness (see [19] for a review). The consciousness definitions used from previous studies in fishes fall between the sleep/awake states and the phenomenal conception or qualia [22]. The definition used by Cabanac *et al.* [22] in his review about the emergence of consciousness in phylogeny was based in Bering & Borklund [23], and it was defined as ‘a higher order cognitive system enabling access to intentional state’. So Cabanac *et al.* proposed that ‘a common mental pathway’ characterizes consciousness and that uses pleasure (or displeasure) as means to optimize behaviour.

One trait that has been identified as indicative of sentience and consciousness is the capacity for showing stress-induced hyperthermia (SIH) or emotional fever [22,24]. These terms refer to a temporary increase in body temperature of about 1–2°C shown in response to a variety of stressors. SIH or emotional fever is a true fever, and the same internal pathways that activate behavioural fever are activated, but the trigger in this case is not an exogenous or endogenous pyrogen (infection by a bacteria or a virus, endotoxin, external or internal lesion, for example) but a stressful or challenging situation (anxiety, novel environment, handling, etc.) [25–27]. For example, catching, handling and restraining procedures have been found to produce SIH in a wide range of birds and mammals, including humans [28–36]. Handling and exposure to a novel environment has also been shown to produce emotional fever induced by behavioural changes in ectothermic vertebrates such as *Callopistes maculatus* lizards [37]. The extent of the SIH response is correlated with the stressor intensity [38,39], making this, for example, a valuable tool for detecting short-term acute stress in welfare research or for the mitigation of chronic, long-term, stressful situations. In adaptive terms, emotional fever contributes to effective fight or flight responses, occurring in parallel with release of energy sources and increases in heart rate [40].

The importance of emotional fever in the debate about fish sentience and consciousness lies in the fact that earlier studies failed to identify SIH in fishes (goldfish: [41]) or amphibians (toads: [42]), even though these animals show well-developed physiological responses to the stressor used (e.g. handling in goldfish [43] and toads [44]). On the basis of these findings, it was concluded that consciousness, and with it the ability to experience emotional fever, evolved exclusively in the amniote lineage and is absent in fishes and amphibians [22]. Cabanac's views are still influential [18–20].

With this background, the aim of this study was to ask again the question of whether fishes have the capacity to show emotional fever. We used zebrafish held in an experimental tank that allowed fish to move freely through a gradient of temperatures centred on the optimal temperature for this species. This novel experimental set-up provides accurate, dynamic information on temperature preferences in zebrafish and has recently been used to demonstrate that behavioural fever following exposure to a virus protects zebrafish by optimizing the antiviral response at the level of the transcriptome [45]. In the present context, the thermal gradient allowed the fish to choose their preferred temperature both undisturbed or after a period of exposure to well-known stressors for fishes [46,47].

## 2. Material and methods

### (a) Animals

Healthy adult zebrafish (*Danio rerio*; 0.93 ± 0.22 g mean weight; 43.73 ± 2.44 mm total length; *n* = 206) were bred in the zebrafish facilities at the Universitat Autònoma de Barcelona, Spain. Zebrafish were originally purchased, as juveniles, from a commercial supplier (Piscicultura Superior s.l., Barcelona, Spain) and held in a recirculating rack system developed by zfbiolabs^®^ (www.zfbiolabs.com). Fish were kept in standard zebrafish housing conditions: 28 ± 0.74°C on a 12 L : 12 D photoperiod cycle [48], and fed twice a day, with a maintenance ratio of about 0.5% body weight per day, on a wet commercial diet developed by zfbiolabs. Water quality indicators (dissolved oxygen, pH, ammonia, nitrite, nitrate and chloride) were analysed periodically (OxiGuard^®^ and SERA tests^®^) and always found under recommended levels [48].

### (b) Experimental tank

The experimental thermal gradient tank was 180 m^3^ (210 × 30 × 30 cm of size) divided with five transparent Plexiglas screens to create six equal interconnected chambers, as used by Boltaña *et al.* [37]. Each screen had a hole in the centre (3 cm diameter; 10 cm from the bottom) linking adjacent chambers and each chamber was held within a different thermal range. Details of the thermal gradient are: chamber 1: 17.92 ± 0.2°C, chamber 2: 24.83 ± 0.26°C, chamber 3: 26.92 ± 0.2°C, chamber 4: 28.75 ± 0.27°C, chamber 5: 32°C and finally chamber 6: 35°C. During an experiment, temperatures for each chamber were recorded for 10 s every 30 min throughout daylight hours. Chambers 1 and 6 were extreme thermal conditions (17.92 ± 0.2°C and 35°C, respectively), convenient for our thermostatic purposes. Chambers 3 and 4 were considered normothermic (26.92 ± 0.2°C and 28.75 ± 0.27°C, respectively, in the ranges of the acclimation temperature in normal zebrafish holding facilities (26–28°C). The regimes in chambers 5 and 6 (32–35°C) were designed to offer hyperthermia or febrile temperatures. Thermal gradients were achieved before the fish acclimation period (see below) with a mean difference in temperature of 17°C between chambers 1 and 6 by simultaneously heating chamber 6 (mean temperature: 35°C) and cooling chamber 1 (mean temperature: 17.92°C). Oxygen concentration and temperatures were recorded several times each day at the same time of the day until gradients were stabilized (see the electronic supplementary material, figure S1) and then they were checked before and after running the experiment each day. Design of the tank was such that oxygen levels were uniformly high in all chambers, in spite of differences in temperature. Water from the tanks was changed for every zebrafish group at the end of the each trial.

### (c) Exposure of fish to the temperature gradients

Fish were housed in the laboratory at the standard husbandry temperature of at 28 ± 0.74°C and so were acclimated to this temperature (for more information about temperature preference in wild zebrafish, laboratory holding and rearing temperatures, see [48]). During the experiment, six groups of fish (*n* = 12 for each group) were introduced into chamber 4 (28.75 ± 0.27 °C) in the evening for acclimation and filming began at 6.00 the next day, providing a minimum of 12 h acclimation in the gradient tank during which the fish could freely choose their preferred temperature.

### (d) Experimental treatments

Three of the six groups were placed in the temperature gradient tank the day before to acclimatize (min 12 h, overnight) and confinement experiments started always at the same time every morning (10.00), at least 4 h after the lights were turned on. Fish from the confinement group were gently captured from the temperature gradient tank where they had acclimatized overnight and were placed into a small fishing net (20 × 14 × 18 cm) hanging inside the water at around 27°C for 15 min (chamber 3). Netting and restraining are known stressors in zebrafish [49]. Following confinement, the fish were gently released again into chamber 3. The remaining three groups (controls) were placed in the temperature gradient tanks unmanipulated and undisturbed, but otherwise received the same treatment. Prior feeding and temperature regimes and all handling procedures were identical for control and experimental fish.

### (e) Behavioural recordings and occupation measurements

Fish were not tagged to avoid extra sources of stress or infection. Scan sampling was used to monitor the distribution of fish within the thermal gradient for a minimum of 8 h after the stressful event in the experimental groups and for an equivalent time in the controls. The distribution of the animals was recorded with three remote control cameras placed each one in front of two chambers, recording for 30 s during each 30 min period for eight diurnal hours (maximum of 108 events recorded; three groups/two conditions). The same observer extracted data from the recordings, after cross validation with an experienced researcher. Every hour recorded was plotted and analysed. The first 4 h were examined for the expression of emotional fever; the remaining 4 h were used to study the dynamics of extinction of the emotional fever response.

### (f) Statistical analysis

As the study had a hierarchical design (repeated measurement on chamber, within group of fish), and a count as the outcome variable, a Poisson response model was constructed within the multilevel modelling statistical software MLwiN v. 2.35 [50]. The hierarchy was specified as above and terms for the treatment, temperature and temperature squared, and measurement occasion (time) and their full interactions tested within the model. Terms were retained if they were significant at *α* ≤ 0.05. An offset was also entered into the model (LnExpected) to accommodate differences in the size of the groups of fish, as one fish in each of one treatment and one control group was lost before the commencement of the study. A Mann–Whitney test to compare the different percentages of fish occupation between controls and confined animals for the three-zebrafish groups on the higher temperature chambers (more than 28°C) were performed. Significance was set up at *p* < 0.05 for all statistical analysis. This statistical analysis was performed with IBM**^®^** SPSS**^®^** 17 Statistics v19 for MAC**^®^** OS X.

## 3. Results

**Figure 1.**
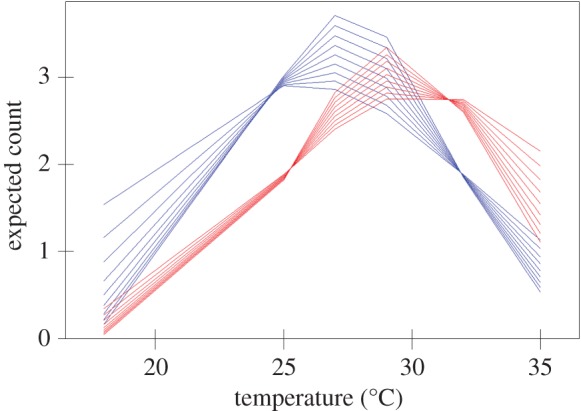
Stress induces hyperthermia in zebrafish under confinement stress. Distribution of zebrafish across the temperature gradient after a confinement stress treatment (red) versus control (blue). The expected mean count is shown for a group of 12 fish. Each line shows the distribution at a different 30 min measurement occasion. The distribution of the confinement treatment groups flattened with increasing time, while that of the control group peaked with increasing time. See the electronic supplementary material figure S2 for further detail.

The full three-way interaction and all lower interactions terms between treatment, temperature and time were significant at *p* < 0.05 and were retained in the model. The parameter estimates and their standard errors for the final model are shown in table 1 and these allow a mathematical model to be constructed that gives the expected Ln (fish count) within a chamber for a given group size, temperature, treatment and time combination. This model is shown graphically in figure 1, which depicts the mean expected count at a given temperature, and time for each of the two treatments (control in blue, confinement in red). Note that the figure shows the back-transformed counts, not the Ln values, and that only details for the groups with *n* = 12 are shown. The groups of 11 show the same patterns of change as the other groups within each treatment, but as their expected counts are adjusted they have been omitted from the figure to retain clarity. It can be seen that the distribution of the confined treatment groups is shifted towards higher temperature chambers. There was a differential effect on the shape of the distributions between the two treatment groups over time—as time increased the confinement groups tended to move from the central chambers to the extremes, while the control groups tended to move in towards the central chambers, from the extremes, over time. This is shown in more detail in the electronic supplementary material, figure S2.

**Table 1.** Model estimates with standard error (s.e.) for the final model.

	model estimates	s.e.
constant	−7.83143	4.3971
LnExpected	2.50374	2.54663
temperature	0.55854	0.30931
temperature sqrd	−0.01088	0.0058
confinement	−21.4824	8.74087
confinement ×temperature	1.40936	0.62066
confinement ×temperature sqrd	−0.02275	0.01094
time	−0.07855	0.02657
time ×temperature	0.00563	0.00193
time ×temperature sqrd	−0.0001	0.00003
time ×confinement	0.14533	0.04813
time ×confinement ×temperature	−0.01039	0.00336
time ×confinement ×temperature sqrd	0.00018	0.00006

**Figure 2.**
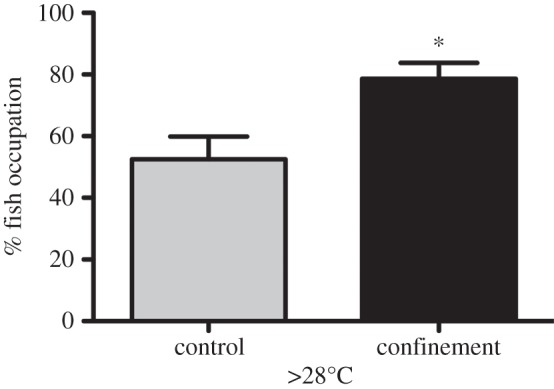
Comparison between control and confined zebrafish in the top half of the temperature gradient (more than 28°C). Fish under confinement stress occupied higher temperatures than control, non-stressed fish (Mann–Whitney test; *p* < 0.05) for the first 4 h after the stressful event. Grey bars represent control groups and black are stress-confined groups (*n* = 6; mean + s.e.m.).

**Figure 3.**
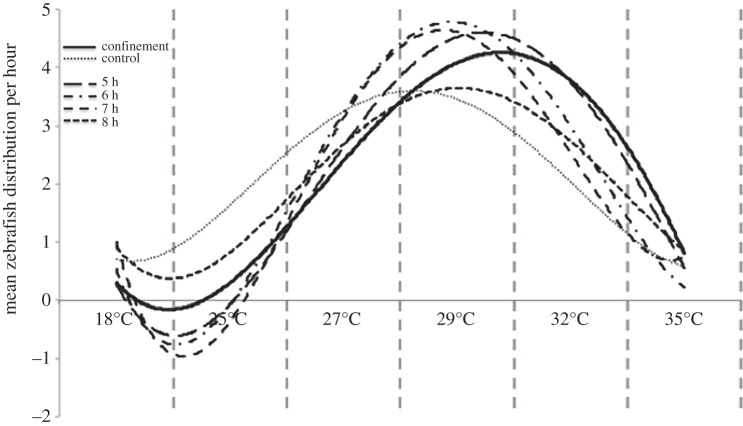
Per hour extinction of the SIH in zebrafish after confinement stress. Polynomial line fitting for zebrafish distribution at the 5th, 6th, 7th and 8th hour after the confinement stress. Zebrafish groups slowly recover their normal distribution in the gradient tank.

All groups of zebrafish under confinement also had a greater percentage of occupation of tank areas at more than 28°C for the first 4 h than control groups (Mann–Whitney test; *p* < 0.05; figure 2). Four hours after the confinement event, a progressive shift to a lower preferred temperatures was observed. Distribution values, similar to control groups, were obtained 8 h after the stressor was applied (fitted polynomial curves for the distribution each hour are depicted in figure 3).

## 4. Discussion

The results of the present experiment show that handling followed by confinement alters temperature preferences in zebrafish, inducing them to spend more time in warmer water, raising their body temperature by an estimated 2–4°C. Cabanac & Laberge [32], differently from our approach, monitored space use in goldfish in a tank with two communicating, differently thermostated, chambers both at higher temperatures (34°C and 37°C) than the thermopreferendum of this species (26–30°C). Though the fish were acclimatized for a week to these higher temperatures, it is not clear how this might have affected them. However, it is possible that having been held at higher than normal temperature may have compromised expression of SIH. Cabanac's failure to demonstrate SIH or emotional fever in fish and his conclusions from this about sentience and consciousness in fish [22,51], remain influential. For example, Allen [19] cites these papers as one of the most relevant contributions to the field.

The provision of the opportunity to move freely within a temperature gradient centred on the species' preferred temperature, as opposed to offering a choice between two relatively high temperatures [41], seems to be critical for demonstrating emotional fever. In the present set-up, with the fish free to make fine-scale behavioural adjustments, such adjustments take the form of emotional fever. This is supported by an unexpected finding in a previous study of behavioural fever induced by viral infection in zebrafish housed in the same temperature gradient tank [45]. Though not designed for this purpose, in this study too, zebrafish that had been stressed (by handling and control saline injection) spent significantly more time in the warmer chambers than did undisturbed controls, though to a lesser extent than those that had been given a viral infection (electronic supplementary material, figure S3). Thus, by quantifying the movement of zebrafish within a thermal gradient we were able to demonstrate the occurrence of SIH, or emotional fever in fish. Individual fish behaviour could not be monitored because fish were not individually tagged or identified, so the current results only refer to the fish as a group of individuals. Tagging was not considered because of the after tagging inflammatory effect and because of the external or internal injuries caused by the tagging that could be interfering with the experiment. Individual differences in response could not be monitored for this experiment but between groups distributions were measured and a significant group effect was observed. Further experiments with fishes individually identified (not by tagging but with a specific tracking software) will be very interesting for a more fine description of the individual fish response to emotional fever behaviour in groups. However, the question in the case of this experiment was just to see if there was a response or not to the confinement stress and not if all individual fish had the same level of response. Obviously and taking into account recent studies, individual fish respond to different levels of intensity for the same stressful event and in this case it will be probably the same case. In fact looking at the results we can see a range of variability on the response to the emotional fever, as it is common for any behaviour and physiological responses.

The fact that fish respond to the stressful event immediately but takes a long time (between 4 and 8 h) to return to normal thermal preference distribution is indicative of how intense stressful situations affect the behaviour of this species. In other stress experiments applied to this species maybe this effect gets masked by the design of the tanks but in this case where they can have proper time and conditions to express their behaviour we can see how difficult it is for them to recover allostasis and return to normal conditions after the stressful event.

Considerable research effort has been aimed at identifying the physiological and molecular mechanisms that underlie SIH; these have shown that it is a real fever in the sense that it activates the same exact physiological and metabolic pathways [52–55]. The underpinning physiological regulation of body temperature in endotherms has been well documented and equivalent structures identified in ectothermic vertebrates including fishes (for review, see [56]). Convergent signalling from the periphery by pyrogenic mediators such as prostaglandin E2 (PGE_2_) targets the preoptical area in the anterior hypothalamus and drives internally generated increases of temperature in endotherms [57]. By contrast, mobile ectotherms regulate body temperature by thermotaxis in order to finely tune physiological responses and increase survival and fitness. In support for a similar underlying circuitry in fishes, behavioural fever in zebrafish under viral challenge promotes increased PGE_2_ levels in the plasma leading to a change in temperature preference and recovery from infection [45]. However, the underpinning mechanism driving thermotaxis is not well understood in fishes. A recent study in *Drosophila* larvae identified thermosensory neurons that drive thermotaxis in a fluctuating thermal environment providing a basis for thermosensory input coupled to environmental navigation [58]. This may provide an interesting avenue to explore how emotional fever is expressed in ectothermic vertebrates.

Our results suggest that, in fishes as in mammals (e.g. laboratory mice; see [40]), changes in thermal preference could potentially be used as a non-invasive operational welfare indicator, In addition, as movement into warmer water in response to stress is likely to be part of the natural process whereby fishes prepare physiologically for imminent challenge, in laboratory and farming conditions, development could be potentially be optimized and welfare improved by provision of thermal gradients at key developmental stages or in advance of predictable stressful circumstances (e.g. challenge, vaccination or transport). By contrast, lack of thermal choice in fishes (whether due to aquaculture practice or, for wild fishes, to climate change) probably contributes to increased stress due to the decreased efficacy of regulatory physiological responses.

## 5. Conclusion

Whatever the underlying mechanisms and associated benefits of the phenomenon and notwithstanding the complex relationship between emotion and consciousness, the fact that the fish used in this study are capable of SIH, or emotional fever, means that the absence of this ability cannot be used to argue for a lack of consciousness in this taxonomic group, as proposed by Cabanac *et al.* [22]. As discussed above, there is a growing body of information from other sources (reviewed by [5–7]) that at least some of the brain mechanisms involved with feeling and emotion in mammals are conserved vertebrate features, that the responses of fishes to noxious stimuli are complex and include a motivational/attentional component and that fishes have well-developed learning capacities and show complex behaviour. Our results add to the emerging picture of fishes as behaviourally complex animals that may well be sentient and conscious to an extent at least. They therefore have important implications both for how the welfare of fishes is conceptualized and protected and for our understanding of the evolution of emotions and consciousness in vertebrates.

## Supplementary Material

SFig1. SFig2. SFig3.

## References

[1] Rose JD 2002 The neurobehavioral nature of fishes and the question of awareness and pain. Rev. Fish. Sci. 10, 1–38. (10.1080/20026491051668)

[2] Arlinghaus R, Cooke SJ, Lyman J, Policansky D, Schwab A, Suski C, Sutton SG, Thorstad EB 2007 Understanding the complexity of catch-and-release in recreational fishing: an integrative synthesis of global knowledge from historical, ethical, social, and biological perspectives. Rev. Fish. Sci. 15, 75–167. (10.1080/10641260601149432)

[3] Rose JD, Arlinghaus R, Cooke SJ, Diggles BK, Sawynok W, Stevens ED, Wynne CDL 2014 Can fish really feel pain? Fish Fish. 15, 97–133. (10.1111/faf.12010)

[4] Chandroo K, Duncan IJ, Moccia R 2004 Can fish suffer? perspectives on sentience, pain, fear and stress. Appl. Anim. Behav. Sci. 86, 225–250. (10.1016/j.applanim.2004.02.004)

[5] Huntingford FA, Adams C, Braithwaite VA, Kadri S, Pottinger TG, Sandoe P, Turnbull JF 2006 Current issues in fish welfare. J. Fish Biol. 68, 332–372. (10.1111/j.0022-1112.2006.001046.x)

[6] Braithwaite V 2010 Do fish feel pain? Oxford, UK: Oxford University Press.

[7] Brown C 2015 Fish intelligence, sentience and ethics. Anim. Cogn. 18, 1–17. (10.1007/s10071-014-0761-0) 24942105

[8] Portavella M, Torres B, Salas C 2004 Avoidance response in goldfish: emotional and temporal involvement of medial and lateral telencephalic pallium. J. Neurosci. 24, 2335–2342. (10.1523/JNEUROSCI.4930-03.2004) 14999085 PMC6730421

[9] Silva PIM, Martins CIM, Khan UW, Gjøen HM, Øverli Ø, Höglund E 2015 Stress and fear responses in the teleost pallium. Physiol. Behav. 141, 17–22. (10.1016/j.physbeh.2014.12.020) 25497079

[10] Sneddon LU, Braithwaite VA, Gentle MJ 2003 Do fishes have nociceptors? Evidence for the evolution of a vertebrate sensory system. Proc. R. Soc. Lond. B 270, 1115–1121. (10.1098/rspb.2003.2349) PMC1691351 12816648

[11] Sneddon LU 2003 The evidence for pain in fish: the use of morphine as an analgesic. Appl. Anim. Behav. Sci. 83, 153–162. (10.1016/S0168-1591(03)00113-8)

[12] Brown C 2012 Tool use in fishes. Fish Fish. 13, 105–115. (10.1111/j.1467-2979.2011.00451.x)

[13] Reese ES 1989 Orientation behaviour of butterflyfishes on coral reefs: spatial learning of route specific landmarks and cognitive maps. In Developments in environmental biology of fishes, vol. 9, pp. 79–86. The Netherlands: Springer.

[14] Burt de Perera T 2004 Fish can encode order in their spatial map. Proc. R. Soc. Lond. B 271, 2131–2134. (10.1098/rspb.2004.2867) PMC1691836 15475332

[15] Grosenick L, Clement TS, Fernald RD 2007 Fish can infer social rank by observation alone. Nature 445, 429–432. (10.1038/nature05646) 17251980

[16] Paśko Ł 2010 Tool-like behavior in the sixbar wrasse, *Thalassoma hardwicke* (Bennett, 1830). Zoo Biol. 29, 767–773. (10.1002/zoo.20307) 20095003

[17] Millot S, Nilsson J, Fosseidengen JE, Bégout M-L, Fernö A, Braithwaite VA, Kristiansen TS 2014 Innovative behaviour in fish: Atlantic cod can learn to use an external tag to manipulate a self-feeder. Anim. Cogn. 17, 779–785. (10.1007/s10071-013-0710-3) 24249160

[18] Anderson DJ, Adolphs R 2014 A framework for studying emotions across species. Cell 157, 187–200. (10.1016/j.cell.2014.03.003) 24679535 PMC4098837

[19] Allen C 2013 Fish cognition and consciousness. J. Agric. Environ. Ethics 26, 25–39. (10.1007/s10806-011-9364-9)

[20] Braithwaite VA, Huntingford F, van den Bos R 2013 Variation in emotion and cognition among fishes. J. Agric. Environ. Ethics 26, 7–23. (10.1007/s10806-011-9355-x)

[21] Ebbesson LOE, Braithwaite VA 2012 Environmental effects on fish neural plasticity and cognition. J. Fish Biol. 81, 2151–2174. (10.1111/j.1095-8649.2012.03486.x) 23252732

[22] Cabanac M, Cabanac AJ, Parent A 2009 The emergence of consciousness in phylogeny. Behav. Brain Res. 198, 267–272. (10.1016/j.bbr.2008.11.028) 19095011

[23] Zelazo PD, Moscovitch M, Thompson E 2007 The Cambridge handbook of consciousness. Cogn. Sci. 39, 981 (10.1163/156916208X311665)

[24] Briese E, De Quijada MG 1970 Colonic temperature of rats during handling. Acta Physiol. Latino Amer. 20, 97–102. 5529391

[25] Bicego KC, Barros RCH, Branco LGS 2007 Physiology of temperature regulation: comparative aspects. Comp. Biochem. Physiol. A. Mol. Integr. Physiol. 147, 616–639. (10.1016/j.cbpa.2006.06.032) 16950637

[26] Singer R, Harker CT, Vander AJ, Kluger MJ 1986 Hyperthermia induced by open-field stress is blocked by salicylate. Physiol. Behav. 36, 1179–1182. (10.1016/0031-9384(86)90497-X) 3725924

[27] Kluger MJ, O'Reilly B, Shope TR, Vander AJ 1987 Further evidence that stress hyperthermia is a fever. Physiol. Behav. 39, 763–766. (10.1016/0031-9384(87)90263-0) 3602130

[28] Cabanac M, Aizawa S 2000 Fever and tachycardia in a bird (*Gallus domesticus*) after simple handling. Physiol. Behav. 69, 541–545. (10.1016/S0031-9384(00)00227-4) 10913794

[29] Cabanac AJ, Guillemette M 2001 Temperature and heart rate as stress indicators of handled common eider. Physiol. Behav. 74, 475–479. (10.1016/S0031-9384(01)00586-8) 11790407

[30] Carere C, Oers VK 2004 Shy and bold great tits (*Parus major*): body temperature and breath rate in response to handling stress. Physiol. Behav. 82, 905–912. (10.1016/S0031-9384(04)00312-9) 15451657

[31] Briese E 1992 Cold increases and warmth diminishes stress-induced rise of colonic temperature in rats. Physiol. Behav. 51, 881–883. (10.1016/0031-9384(92)90130-T) 1594688

[32] Briese E 1995 Emotional hyperthermia and performance in humans. Physiol. Behav. 58, 615–618. (10.1016/0031-9384(95)00091-v) 8587973

[33] Korhonen H, Niemelä P, Jauhiainen L, Tupasela T 2000 Effects of space allowance and earthen floor on welfare-related physiological and behavioural responses in male blue foxes. Physiol. Behav. 69, 571–580. (10.1016/S0031-9384(00)00215-8) 10913798

[34] Gray DA, Maloney SK, Kamerman PR 2008 Restraint increases afebrile body temperature but attenuates fever in Pekin ducks (*Anas platyrhynchos*). Amer. J. Physiol. Regul. Integr. Comp. Physiol. 294, R1666–R1671. (10.1152/ajpregu.00865.2007) 18337310

[35] Meyer LCR, Fick L, Matthee A, Mitchell D, Fuller A 2008 Hyperthermia in captured impala (*Aepyceros melampus*): a fright not flight response. J. Wildl. Dis. 44, 404–416. (10.7589/0090-3558-44.2.404) 18436672

[36] Busnardo C, Tavares RF, Resstel LBM, Elias LLK, Correa FMA 2010 Paraventricular nucleus modulates autonomic and neuroendocrine responses to acute restraint stress in rats. Auton. Neurosci. Basic Clin. 158, 51–57. (10.1016/j.autneu.2010.06.003) 20594922

[37] Cabanac AJ, Gosselin F 1993 Emotional fever in the lizard *Callopistes maculatus*. Anim. Behav. 46, 200–202. (10.1006/anbe.1993.1178)

[38] Vinkers CH, Bogaert MJV, Klanker M, Korte SM, Oosting R, Hanania T, Hopkins SC, Olivier B, Groenink L 2008 Translational aspects of pharmacological research into anxiety disorders: the stress-induced hyperthermia (SIH) paradigm. Eur. J. Pharmacol. 585, 407–425. (10.1016/j.ejphar.2008.02.097) 18420191

[39] Bhatnagar S, Vining C, Iyer V, Kinni V 2006 Changes in hypothalamic-pituitary-adrenal function, body temperature, body weight and food intake with repeated social stress exposure in rats. J. Neuroendocrinol. 18, 13–24. (10.1111/j.1365-2826.2005.01375.x) 16451216

[40] Adriaan Bouwknecht J, Olivier B, Paylor RE 2007 The stress-induced hyperthermia paradigm as a physiological animal model for anxiety: a review of pharmacological and genetic studies in the mouse. Neurosci. Biobehav. Rev. 31, 41–59. (10.1016/j.neubiorev.2006.02.002) 16618509

[41] Cabanac M, Laberge F 1998 Fever in goldfish is induced by pyrogens but not by handling. Physiol. Behav. 63, 377–379. (10.1016/S0031-9384(97)00444-7) 9469730

[42] Cabanac AJ, Cabanac M 2004 No emotional fever in toads. J. Thermal Biol. 29, 669–673. (10.1016/j.jtherbio.2004.08.039)

[43] Dror M, Sinyakov MS, Okun E, Dym M, Sredni B, Avtalion RR 2006 Experimental handling stress as infection-facilitating factor for the goldfish ulcerative disease. Vet. Immunol. Immunopathol. 109, 279–287. (10.1016/j.vetimm.2005.08.022) 16174536

[44] Narayan EJ, Cockrem JF, Hero J-M 2013 Are baseline and short-term corticosterone stress responses in free-living amphibians repeatable? *Comparative biochemistry and physiology**.* Part A Mol. Integr. Physiol. 164, 21–28. (10.1016/j.cbpa.2012.10.001) 23047053

[45] Boltaña S et al. 2013 Behavioural fever is a synergic signal amplifying the innate immune response. Proc. R. Soc. B 280, 20131381 (10.1098/rspb.2013.1381) PMC3730603 23843398

[46] Foo JTW, Lam TJ 1993 Serum cortisol response to handling stress and the effect of cortisol implantation on testosterone level in the tilapia, *Oreochromis mossambicus*. Aquaculture 115, 145–158. (10.1016/0044-8486(93)90365-6)

[47] Galhardo L, Vital J, Oliveira RF 2011 The role of predictability in the stress response of a cichlid fish. Physiol. Behav. 102, 367–372. (10.1016/j.physbeh.2010.11.035) 21145905

[48] Lawrence C 2007 The husbandry of zebrafish (*Danio rerio*): a review. Aquaculture 269, 1–20. (10.1016/j.aquaculture.2007.04.077)

[49] Ramsay JM, Feist GW, Varga ZM, Westerfield M, Kent ML, Schreck CB 2009 Whole-body cortisol response of zebrafish to acute net handling stress. Aquaculture 297, 157–162. (10.1016/j.aquaculture.2009.08.035) 25587201 PMC4289633

[50] Rasbash J, Charlton C, Browne WJ, Healy M, Cameron B 2009 MLwiN, version 2.1. Bristol, UK: Centre for Multilevel Modelling, University of Bristol.

[51] Cabanac M 1999 Emotion and phylogeny. Jpn J. Physiol. 49, 1–10. (10.2170/jjphysiol.49.1) 10219103

[52] Kluger MJ, Kozak W, Conn CA, Leon LR, Soszynski D 1996 The adaptive value of fever. Infect. Dis. Clin. North Amer. 10, 1–20. (10.1016/S0891-5520(05)70282-8) 8698984

[53] Briese E, Cabanac M 1991 Stress hyperthermia: physiological arguments that it is a fever. Physiol. Behav. 49, 1153–1157. (10.1016/0031-9384(91)90343-M) 1896496

[54] Soszynski D 2006 Molecular mechanism of emotional fever: the role of nitric oxide. J. Physiol. Pharmacol. Official J. t Polish Physiol. Soc. 57(Suppl 8), 51–59. 17242472

[55] Hamada FN, Rosenzweig M, Kang K, Pulver SR, Ghezzi A, Jegla TJ, Garrity PA 2008 An internal thermal sensor controlling temperature preference in *Drosophila*. Nature 454, 217–220. (10.1038/nature07001) 18548007 PMC2730888

[56] Nakamura K 2011 Central circuitries for body temperature regulation and fever. Am. J. Physiol. Regul. Integr. Comp. Physiol. 301, R1207–R1228. (10.1152/ajpregu.00109.2011) 21900642

[57] Boulant JA 2000 Role of the preoptic-anterior hypothalamus in thermoregulation and fever. Clin. Infect. Dis. Official Public. Infect. Dis. Soc. Amer. 31(Suppl 5), S157–S161. (10.1086/317521) 11113018

[58] Klein M et al. 2014 Sensory determinants of behavioral dynamics in *Drosophila* thermotaxis. Proc Natl Acad. Sci. USA 112, E220–E229. (10.1073/pnas.1416212112) 25550513 PMC4299240

