# Supplementary material for: Fish can show emotional fever: stress-induced hyperthermia in zebrafish
Source: Proc Biol Sci. 2015 Nov 22;282(1819):20152266. doi: 10.1098/rspb.2015.2266 (PMC4685827; doi:10.1098/rspb.2015.2266)
Supplement: SFig1. SFig2. SFig3. [file rspb20152266supp1.pdf]

Supplementary figures

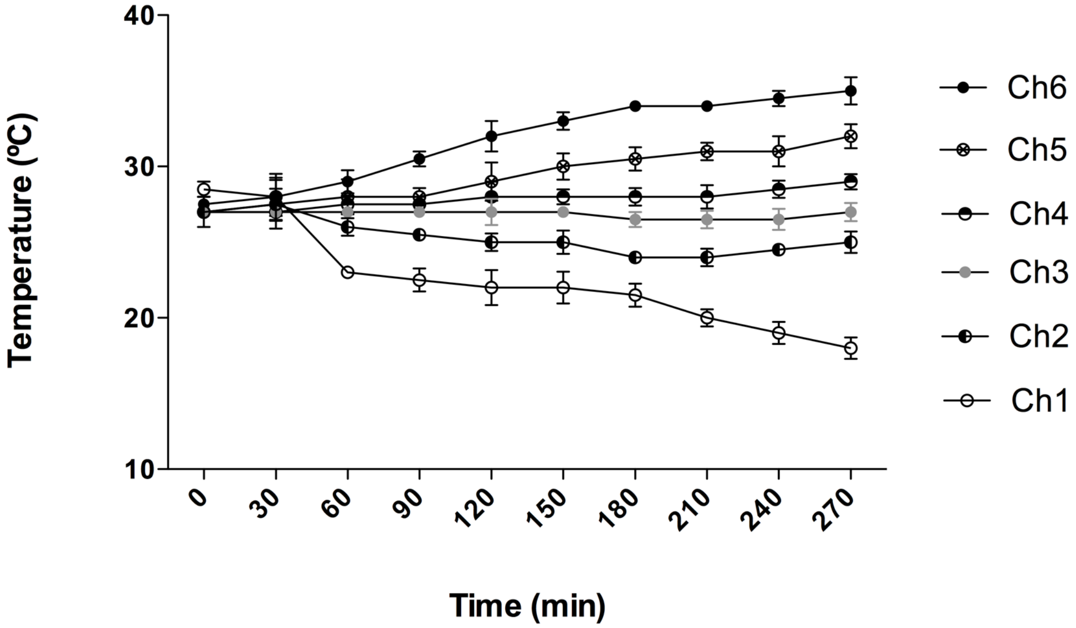

SFigure 1. Gradient tank establishment. After 4 hours the gradient established and remained constant for the time of the experiment. Final chamber temperatures were chamber 1: 17.92±0.2 °C, Chamber 2: 24.83±0.26 °C, Chamber 3: 26.92±0.2 °C, Chamber 4: 28.75 ±0.27 °C, Chamber 5: 32°C and finally Chamber 6: 35°C.

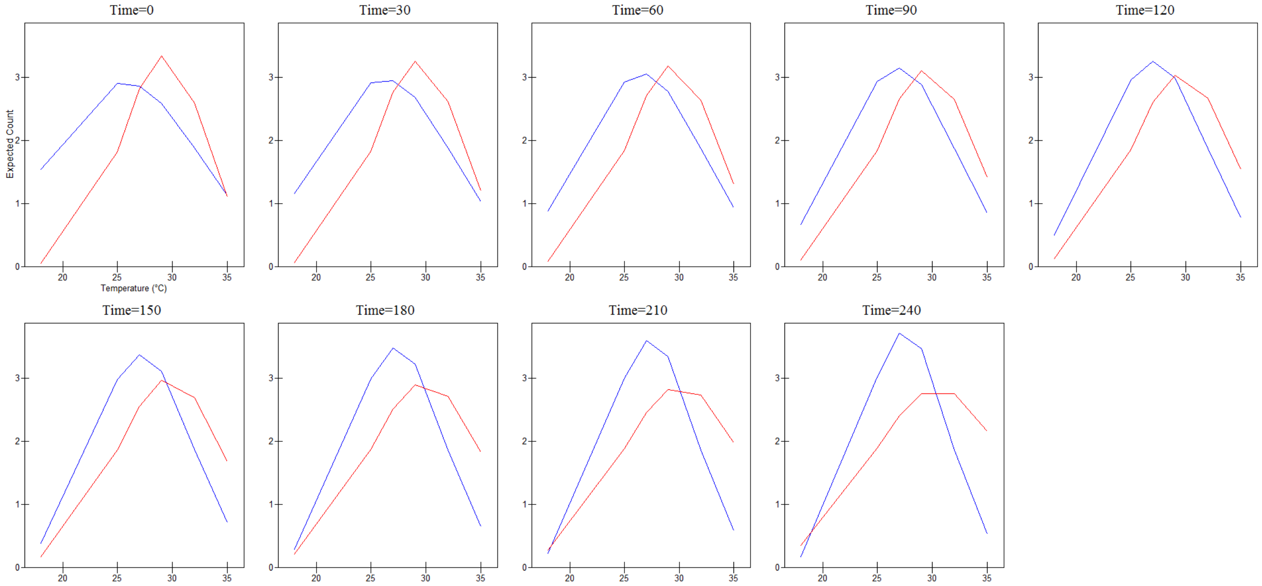

SFigure 2. Detail of the changes in distribution of the groups across the temperature gradient over time. The distributions are shown for each of the two treatments (control in blue, confinement in red) at each of the 30 min sampling times.

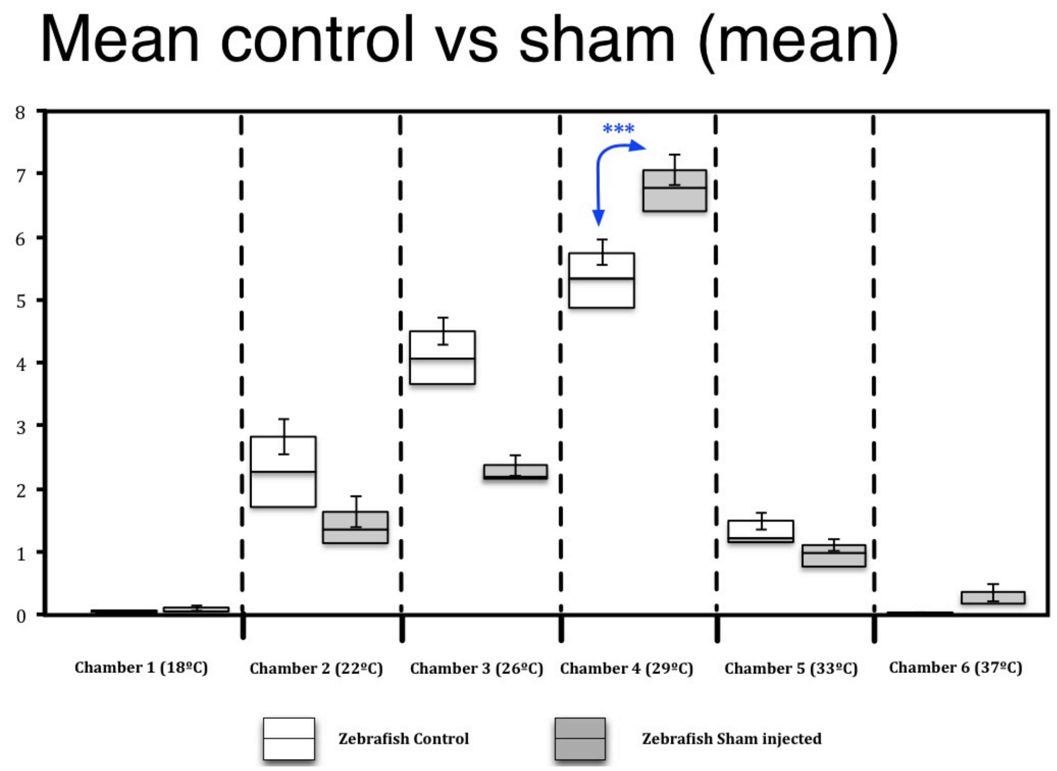

SFigure 3. Holding and PBS injection as a stress factor to induce hyperthermia in Zebrafish. Distribution of zebrafish per chamber of the thermal gradient tank after handling and i.p. injection .
